# Protozoan ALKBH8 Oxygenases Display both DNA Repair and tRNA Modification Activities

**DOI:** 10.1371/journal.pone.0098729

**Published:** 2014-06-10

**Authors:** Daria Zdżalik, Cathrine B. Vågbø, Finn Kirpekar, Erna Davydova, Alicja Puścian, Agnieszka M. Maciejewska, Hans E. Krokan, Arne Klungland, Barbara Tudek, Erwin van den Born, Pål Ø. Falnes

**Affiliations:** 1 Department of Biosciences, University of Oslo, Oslo, Norway; 2 Institute of Genetics and Biotechnology, University of Warsaw, Warsaw, Poland; 3 Department of Cancer Research and Molecular Medicine, Norwegian University of Science and Technology, Trondheim, Norway; 4 Department of Biochemistry and Molecular Biology, University of Southern Denmark, Odense, Denmark; 5 Department of Neurophysiology, Nencki Institute of Experimental Biology, Polish Academy of Sciences, Warsaw, Poland; 6 Institute of Biochemistry and Biophysics, Polish Academy of Sciences, Warsaw, Poland; 7 Clinic for Diagnostics and Intervention and Institute of Medical Microbiology, Oslo University Hospital, Rikshospitalet, Oslo, Norway; 8 Institute of Basic Medical Sciences, University of Oslo, Oslo, Norway; Universität Stuttgart, Germany

## Abstract

The ALKBH family of Fe(II) and 2-oxoglutarate dependent oxygenases comprises enzymes that display sequence homology to AlkB from *E. coli*, a DNA repair enzyme that uses an oxidative mechanism to dealkylate methyl and etheno adducts on the nucleobases. Humans have nine different ALKBH proteins, ALKBH1–8 and FTO. Mammalian and plant ALKBH8 are tRNA hydroxylases targeting 5-methoxycarbonylmethyl-modified uridine (mcm^5^U) at the wobble position of tRNA^Gly(UCC)^. In contrast, the genomes of some bacteria encode a protein with strong sequence homology to ALKBH8, and robust DNA repair activity was previously demonstrated for one such protein. To further explore this apparent functional duality of the ALKBH8 proteins, we have here enzymatically characterized a panel of such proteins, originating from bacteria, protozoa and mimivirus. All the enzymes showed DNA repair activity *in vitro*, but, interestingly, two protozoan ALKBH8s also catalyzed wobble uridine modification of tRNA, thus displaying a dual *in vitro* activity. Also, we found the modification status of tRNA^Gly(UCC)^ to be unaltered in an ALKBH8 deficient mutant of *Agrobacterium tumefaciens*, indicating that bacterial ALKBH8s have a function different from that of their eukaryotic counterparts. The present study provides new insights on the function and evolution of the ALKBH8 family of proteins.

## Introduction

**Figure 1:**
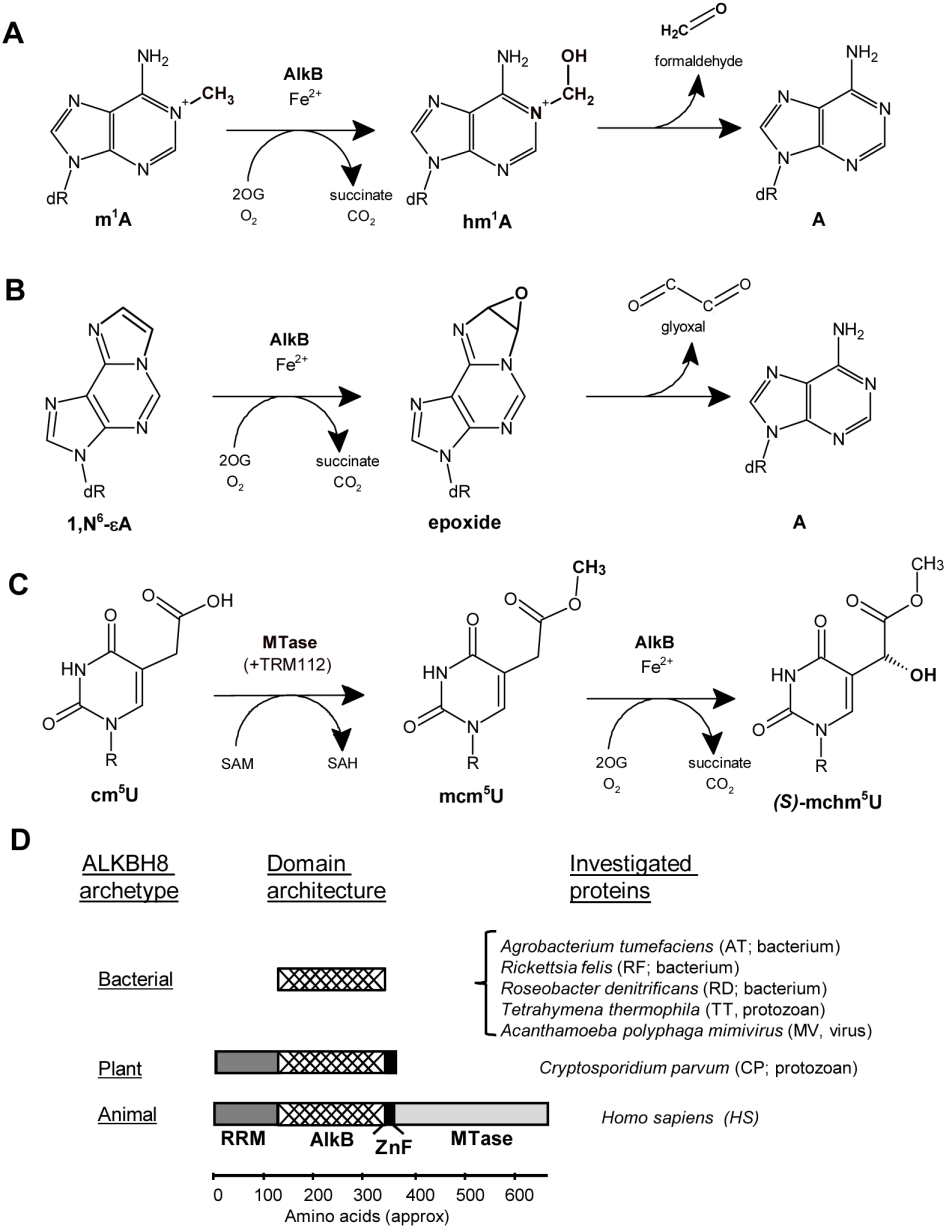
Selected ALKBH8 homologues and modification/repair reactions catalyzed by ALKBH8/AlkB. (A) AlkB-catalyzed DNA repair reaction on the methyl adduct m^1^A. (B) AlkB-catalyzed DNA repair reaction on the etheno adduct 1, *N* ^6^-εA. (C) ALKBH8-catalyzed modification reactions on tRNA. SAM, *S*-adenosylmethionine; SAH, *S*-adenosylhomocysteine. (D) Overview of presently investigated and canonical ALKBH8 proteins and their domain architecture. Gene identification (gi) numbers of the investigated proteins are as follows (size is indicated in parenthesis): AT (195 aa), 159184347; RF (188 aa), 67458989; RD (195 aa), 115345714; TT (199 aa), 118368517; MV (210 aa), 311978317; CP (350 aa), 66362644; HS (664 aa), 195927056.

Over 30 years ago, inactivation of the *alkB* gene of *Escherichia coli* was shown to result in hypersensitivity towards certain methylation agents that target the DNA bases, such as methyl methane sulfonate (MMS) [1]. Correspondingly, *E. coli* AlkB (EcAlkB) was later shown to be a repair enzyme that was able to demethylate the DNA lesions 1-methyladenine and 3-methylcytosine, as well as their structural analogs 1-methylguanine and 3-methylthymine [2]–[6]. AlkB belongs to the superfamily of Fe(II) and 2-oxoglutarate (2OG) dependent dioxygenases, a group of enzymes which require ferrous iron and couple the oxidation of a primary substrate to the decarboxylation of the co-substrate 2OG [7], [8]. In the AlkB reaction, hydroxylation of the deleterious methyl group is followed by spontaneous release of the resulting hydroxymethyl moiety as formaldehyde (Fig. 1A) [3], [6]. EcAlkB is active both on single-stranded (ss) and double-stranded (ds) DNA, and, intriguingly, also on RNA substrates, suggesting a possible role in RNA repair [9]–[11]. In addition to methyl lesions, EcAlkB can also repair bulkier adducts, such as ethyl, hydroxyethyl, propyl and hydroxypropyl groups, as well as exocyclic etheno, ethano, hydroxyethano, and hydroxypropano adducts [12]–[18]. Repair of etheno adduct leads to the release of the etheno moiety as glyoxal (Fig. 1B) [12].

Mammals have nine different AlkB homologues (ALKBH): ALKBH1 to ALKBH8, as well as the fat mass- and obesity-associated protein FTO [7], [19], [20]. *In vitro* and *in vivo* studies indicated that ALKBH2 and ALKBH3 are repair enzymes with a function similar to that of EcAlkB, while non-repair functions have been demonstrated for several other ALKBHs [9], [10], [13], [21], [22]. ALKBH8 was shown to be a bifunctional tRNA modification enzyme (see below), while FTO and ALKBH5 both demethylate the mRNA modification N^6^-methyladenine [23], [24]. Knock-out mice lacking ALKBH1 or ALKBH4 displayed elevated levels of methylation on specific lysine residues in histone 2B or actin, respectively, suggesting that these proteins may be lysine-specific protein demethylases [25], [26].

tRNAs from all three kingdoms of life are subject to extensive post-transcriptional modification, and approximately 100 distinctly modified tRNA nucleosides have been identified [27]. Uridine, when present at the wobble position of the anticodon (position 34), is usually modified, and such modification substantially affects tRNA decoding properties [28]. In eukaryotes, wobble uridines are modified to either 5-methoxycarbonylmethyluridine (mcm^5^U), 5-carbamoylmethyluridine (ncm^5^U), or derivatives thereof [29]. In the yeast *Saccharomyces cerevisiae*, the last step in the synthesis of mcm^5^U is mediated by Trm9, a methyltransferase (MTase) catalyzing the methyl-esterification of 5-carboxymethyluridine (cm^5^U) into mcm^5^U [30]. In mammals, this reaction is catalyzed by the Trm9-like, C-terminal MTase domain of ALKBH8, together with the accessory protein TRM112 (Fig. 1C) [31], [32]. Mammalian ALKBH8 also contains an N-proximal AlkB domain flanked by a RNA-recognition motif (RRM) and a cysteine-rich zinc-finger (ZnF) (Fig. 1D). Recently, the RRM/AlkB/ZnF portion of ALKBH8 was shown to hydroxylate mcm^5^U, leading to the formation of wobble (*S*)-5-methoxycarbonylhydroxymethyluridine [(*S*)-mchm^5^U] specifically in tRNA^Gly(UCC)^ (Fig. 1C) [33], [34].

ALKBHs appear to be present in all multicellular eukaryotes and in a wide range of unicellular eukaryotes and prokaryotes [35], [36]. Moreover, AlkB-like proteins are also found in viruses, particularly in RNA viruses that infect plants, and these proteins display repair activities similar to EcAlkB, suggesting that they may be involved in removing methyl lesions from the viral RNA genome [37]. Bacterial AlkB proteins can be subdivided into four groups based on sequence similarity. Three of the groups comprise proteins with similarity to the mammalian repair proteins ALKBH2 and ALKBH3, whereas members of the fourth group are similar to ALKBH8, but lack the RRM, ZnF and MTase domains (Fig. 1D) [38]. Interestingly, when AlkB proteins from the four groups were investigated, the tested proteins all displayed repair activity on DNA. Notably, the tested ALKBH8-like protein, originating from *Rhizobium etli* (*R. etli*), did not show DNA repair activity on methylated bases, but rather on etheno adducts [38]. Plants, such as *Arabidopsis thaliana*, possess putative orthologues of several of the ALKBH proteins found in mammals, including ALKBH8. The plant orthologue of the mammalian ALKBH8 oxygenase contains the RRM/AlkB/ZnF moiety (Fig. 1D), whereas a different gene encodes a Trm9-like MTase. In *Arabidopsis*, these two proteins were shown to represent the functional equivalent of the bifunctional mammalian ALKBH8 [39].

As both repair and modification activities have been demonstrated for ALKBH8 proteins, we have here sought to further illuminate their biological function by investigating such proteins from several different organisms. The proteins were investigated both for DNA repair activity and for the ability to convert mcm^5^U to (*S*)-mchm^5^U in tRNA. We detected *in vitro* DNA repair activity for all tested recombinant proteins, and for two protozoan ALKBH8s we could also detect tRNA modifying activity. To analyse whether bacterial ALKBH8, like its mammalian counterpart, is involved in wobble uridine modification of tRNA^Gly(UCC)^, we also generated an ALKBH8-deficient strain of *Agrobacterium tumefaciens*, and analysed the tRNA^Gly(UCC)^ modification status. We found the wobble uridine modification status of tRNA^Gly(UCC)^ to be unaltered in this ALKBH8-deficient strain, indicating that bacterial ALKBH8s are not involved in modifying this site.

## Materials and Methods

### Bioinformatics Analysis

Protein sequences of putative ALKBH8 homologues were retrieved from BLAST searches and previous publications [34], [38], [40]. Multiple sequence alignments were constructed using the MUSCLE algorithm [41]. Alignments were manually edited in the Jalview package [42].

### Plasmid Construction

Genes encoding ALKBH8 proteins were amplified by polymerase chain reaction (PCR) from genomic DNA in case of bacterial or protozoan AlkB genes, and a plasmid in case of the mimivirus AlkB protein. For expression purposes, all *Tetrahymena thermophila* TAA and TAG codons were changed to CAA and CAG, respectively, by PCR-mediated mutagenesis, since *T. thermophila* uses an alternative genetic code where UAA and UAG (which normally are stop codons) code for glutamine. Primers used for PCR are listed in Table S1. The PCR products were subsequently cloned into the appropriate restriction sites in plasmid pET-28a(+) (Novagen, Darmstadt, Germany), which was used for expression of recombinant protein in *E. coli*. For bacterial reactivation assays AlkB-encoding fragments from the pET-28a(+)-derived plasmids were subcloned to pJB658 using appropriate restriction sites [43]. The construction of plasmids encoding EcAlkB, human ALKBH2, human ALKBH8, or its AlkB domain (RRM-AlkB; aa 1–354), and subsequent protein expression and purification were previously described [9], [32], [38]. To remove the RRM domain from both ALKBH8 and RRM-AlkB, a PCR was performed to obtain the coding regions for AlkB-MTase (aa 129–664) and for the AlkB core (aa 129–338), respectively. To inactivate the Zn-finger, three Zn-coordinating cysteines were mutated to alanine (*i.e.* Cys341Ala, Cys343Ala and Cys349Ala) by fusion PCR. PCR products were placed into the pET28a(+) vector using the NdeI and SalI restriction sites.

### Protein Expression and Purification

Plasmids pET-28a(+) containing sequences coding for N-terminally 6xHis-tagged ALKBH8 proteins were transformed into the *E. coli* strain BL21-CodonPlus(DE3)-RIPL (Stratagene, La Jolla, CA, USA). When the bacterial culture reached an optical density of 1 measured at 600 nm (OD_600_), expression of recombinant proteins was induced by adding isopropyl-beta-D-thiogalactopyranoside (IPTG) to a final concentration of 0.5 mM and incubation was continued at 16°C for 16 hours. Cells were harvested by centrifugation at 5000×g for 10 min at 4°C and resuspended in a buffer containing 50 mM sodium phosphate (pH 7.0), 150 mM NaCl, 5 mM imidazole, 0.01% Tween 20, EDTA-free Complete-Protease inhibitor (Roche), and 5 mM β-mercaptoethanol. The cells were lysed by addition of lysozyme to a final concentration of 1 mg/ml, incubation on ice for 30 min and subsequent sonication with three 11 W pulses of 30 sec with 30 sec intervals. Cell debris was removed by centrifugation at 12,000×g for 10 min at 4°C. The obtained supernatant was directly mixed with TALON Metal Affinity Resin (Clontech, Mountain View, CA, USA) and recombinant proteins were obtained by a single affinity purification step according to the manufacturer’s instructions. Protein purity and yield were assessed by 15% SDS–PAGE followed by coomassie brilliant blue-staining of the gel.

### Bacterial Survival Assay and Phage Reactivation Assay

To test the ability of ALKBH8 proteins for complementation of the repair function of EcAlkB protein, pJB658-derived plasmids encoding these proteins were transformed into the F-pilus-expressing, *alkB*-deficient *E. coli* strain HK82/F’. Protein expression was induced by the addition of 2 mM toluic acid (Fluka/Sigma-Aldrich). To introduce methyl lesions or etheno adducts, ssDNA bacteriophage M13mp18, ssRNA bacteriophage MS2 or bacteria were treated with methyl methanesulphonate (MMS; Sigma-Aldrich) or chloroacetaldehyde (CAA; Sigma-Aldrich), respectively. Phage and bacteria survival was scored by counting the resulting plaques or bacterial colonies, respectively. The experiments were performed essentially as previously described [38].

### Assay for AlkB-mediated Decarboxylation of 2-oxoglutarate

To determine whether the recombinant ALKBH8 proteins can catalyze uncouple decarboxylation of 2OG, we used the method described earlier [37], which measures the level of radioactive [1-^14^C] succinate produced as a result of decarboxylation of [5-^14^C] 2-oxoglutarate.

### *In vitro* DNA Repair Assay

The oligonucleotide substrates containing m^1^A, m^3^C, 1, *N* ^6^-ethenoadenine (1, *N* ^6^-εA) and 3, *N* ^4^-ethenocytosine (3, *N* ^4^-εC) were purchased from Chemgenes Corporation, USA. Repair reactions were performed by incubating 100 pmoles of AlkB protein with 1 pmol of ^32^P-labeled ssDNA or dsDNA oligonucleotides at 37°C for 30 min in a 50 µl reaction mixture containing 50 mM Tris-HCl (pH 8.0), 2 mM ascorbic acid, 1 mM 2-oxoglutarate, and 80 µM (NH_4_)_2_Fe(SO_4_)_2_•6H_2_O. Reactions were stopped by incubation at 65°C for 20 minutes. In order to remove AlkB proteins, the reaction mixtures were incubated with 1 µl of 20 mg/ml proteinase K (Sigma-Aldrich) for 15 min at 42°C, and then proteinase K was heat inactivated at 90°C for 10 minutes. When reactions were performed with ssDNA substrates, the complementary DNA oligonucleotide was added prior to the next step. AlkB-treated oligonucleotides with methyl lesions were incubated with 20 units of *Dpn*II for 1 hour at 37°C [22], whereas the oligonucleotides containing etheno adducts were incubated for 30 min at 37°C with human alkyl*-*N-purine-DNA glycosylase (ANPG) for 1, *N* ^6^-εA or *E. coli* uracil-DNA glycosylase Mug in case of 3, *N* ^4^-εC and 1, *N* ^2^-εG, followed by abasic site cleavage with human AP endonuclease 1 (HAP1) for 30 minutes at 37°C. Reaction products were resolved by 20% denaturing PAGE in the presence of 7 M urea and visualized by phosphorimaging using FLA-7000 screens (Fujifilm). Quantification was performed by using MultiGauge Software (Fujifilm).

### Construction of an *Agrobacterium tumefaciens alkB* Null Mutant

An *Agrobacterium tumefaciens* C58 *alkB* null mutant (*A. tumefaciens* C58 *alkB^−^*) was made by insertion of a group II intron using the TargeTron Gene Knockout System (Sigma Aldrich) according to the provided manual. An *alkB*-targeted intron was generated by PCR using primers designed by using the TargeTron algorithm (SigmaAldrich) (Table S1) [44]. The generated PCR products were cloned into the *Hin*dIII and *Bsr*GI sites of the pBL1 plasmid, which was a kind gift from Dr. Alan Lambowitz [45]. The donor plasmid, pBL1/*alkB*, was transformed into *A. tumefaciens* C58 by electroporation and the bacteria were selected on YEB agar plates containing 2 µg/ml tetracycline and 100 µg/ml ampicillin. For gene targeting, *A. tumefaciens* C58 containing pBL1/*alkB* was grown at 30°C in YEB medium containing 2 µg/ml tetracycline and expression of the TargeTron cassette was induced by adding *m*-toluic acid to a final concentration of 5 mM when the culture reached early log phase (OD_600_ = 0.3 to 0.4) followed by 3 h growth under the same conditions. After induction, cells were selected on YEB agar plates containing 2 µg/ml tetracycline and 100 µg/ml ampicillin, and incubated at 30°C until colonies were formed (2–3 days). Colonies were screened by colony PCR using ALKBH8-specific primers. Bacteria from a single colony containing the TargeTron cassette inserted into the *alkB* gene were cured of the TargeTron donor plasmid by several passages on YEB agar plates with ampicillin, but without tetracycline. Intron insertion was confirmed by sequencing analysis.

### Survival of *Agrobacterium tumefaciens* C58 Wild-type and *alkB* Mutant after MMS Treatment

*A. tumefaciens* C58 wild-type and *alkB* mutant were grown at 30°C in YEB medium with 50 µg/ml ampicillin. As a negative and positive control HK82/F’, *E. coli* containing pJB658 or pJB658-EcAlkB, respectively, were used. The MMS and CAA treatment was done as described above for the bacterial survival assay with the difference that the induction step with toluic acid was omitted for *A. tumefaciens* C58.

### Total tRNA and Isoacceptor Isolation

Total tRNA from *S. cerevisiae* and *E. coli* were purchased from Roche. Total tRNA from *A. tumefaciens* C58 wild-type and *alkB* mutant was purified using an RNA/DNA maxi kit (Qiagen) according to the manual provided. tRNA^Gly(UCC)^ was purified from total tRNA using 3′-biotinylated oligonucleotides (Table S1), as previously described [32].

### Enzymatic Treatment of tRNA

tRNA from *S. cerevisiae* (5 to 10 µg) was incubated with 100 pmoles of recombinant proteins for 30 min at 37°C in a 50 µl reaction mixture containing 50 mM HEPES-KOH pH 7.5, 0.5 mM MgCl_2_, 2 mM ascorbic acid, 100 µM 2-oxoglutarate, 40 µM FeSO_4_, and 10 U RNasin Plus RNase inhibitor (Promega). Reactions were stopped by incubation for 15 min at 42°C with 1 µl of 20 mg/ml protease K (Sigma-Aldrich). tRNA was extracted with 1 volume of acidic phenol pH 4.0 and chloroform, followed by precipitation with 1 volume of isopropanol in the presence of 1 M NH_4_Ac and 10 µg of glycogen. Pellets were washed with 70% EtOH, dried and dissolved in H_2_O. The samples were subjected to nucleoside analysis by LC–MS/MS.

### Mass Spectrometry

LC-MS/MS of nucleosides was performed essentially as described previously [32]. Briefly, tRNA was enzymatically digested to nucleosides [46], which were separated by reverse phase high-performance liquid chromatography, followed by mass spectrometry detection. Quantification was performed by comparison with pure nucleoside standards run in between the samples.

For MALDI-TOF mass spectrometry, tRNA isoacceptors were digested with RNase T1 (Ambion) and samples prepared for MALDI mass spectrometry as previously described [32].

## Results

### Selection and Bioinformatics Analysis of ALKBH8 Proteins from Various Organisms

Previous studies have established a tRNA modification function for ALKBH8 proteins from animals and plants, whereas robust DNA repair activity on etheno lesions was detected for ALKBH8 from the α-proteobacterium *R. etli* [33], [34], [38], [39]. To further address the function of the ALKBH8 proteins, for the present study we selected such proteins from a wide range of organisms for analysis with respect to both tRNA modification and DNA repair capabilities.

Bacterial ALKBH8 proteins are primarily found in α-proteobacteria, and from this group we chose to examine the ones from *Rickettsia felis* (RF), *Roseobacter denitrificans* (RD), and *Agrobacterium tumefaciens* (AT). In fact, the AT protein was investigated by us previously, but we failed to purify soluble recombinant protein [38]. It is included here because it is possible to generate gene knock-outs of the corresponding bacterium by the so-called TargeTron technology [45]. We also included in our study ALKBH8 proteins from the protozoan *Tetrahymena thermophila* (TT) and from the mimivirus *Acanthamoeba polyphaga mimivirus* (MV), which, like the bacterial ALKBHs, lack annotated domains apart from the defining AlkB domain. In addition, we chose to study the ALKBH8 protein from the protozoan *Cryptosporidium parvum* (CP) which, similarly to plant ALKBH8, has an RRM/AlkB/ZnF domain architecture. The selected proteins and their domain architecture are outlined in Fig. 1D.

**Figure 2:**
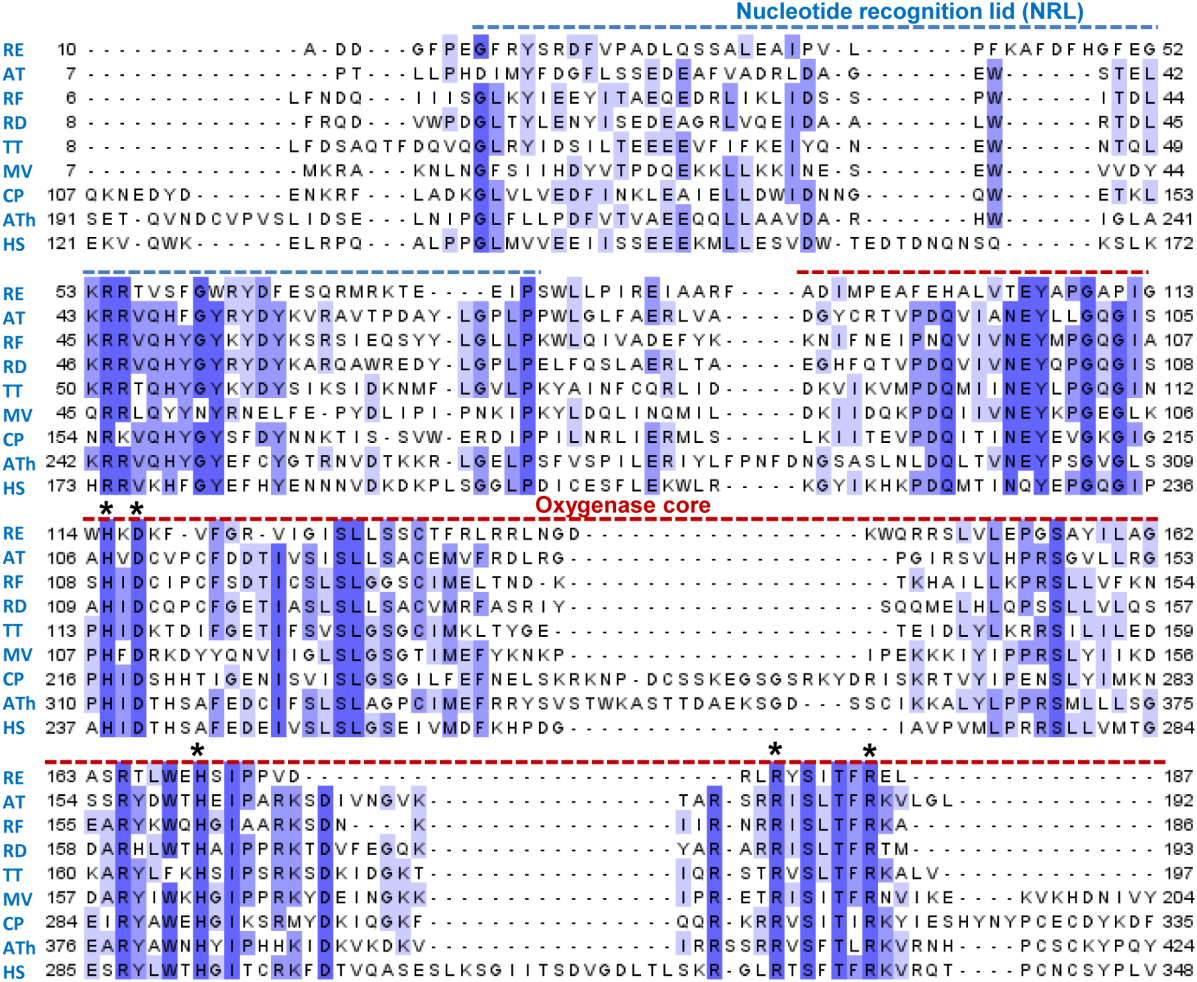
Sequence alignment of ALKBH8 proteins investigated in present study. The ALKBH8 proteins indicated in Fig. 1D were aligned by using the MUSCLE algorithm. For comparison, the previously characterized ALKBH8 proteins from the plant *Arabidopsis thaliana* (Ath; gi|159184347) and the bacterium *Rhizobium etli* (RE; gi|86360251) were also included. The dotted blue line indicates the nucleotide recognition lid, a region implicated in the nucleic acid binding of the EcAlkB protein, whereas the red dotted line indicates the oxygenase core, the region shared between all 2OG/Fe(II) dependent oxygenases. Asterisks indicate the HXDX_n_H triad involved in Fe(II) coordination and the RX_5_R motif characteristic of the ALKBH family of proteins.

To illustrate the degree of sequence similarity between the ALKBH8 proteins, we generated a sequence alignment of the proteins selected for the current study, as well as human and *Arabidopsis* ALKBH8, which have been the focus of previous studies. The alignment shows that these proteins, despite their diverse origins, have a relatively high degree of sequence similarity, both in the core oxygenase region and in the so-called NRL (nucleotide recognition lid) region, which has been implicated in specific binding of the nucleic acid substrate [47]. Moreover, Fe(II)-coordinating residues characteristic of the 2OG/Fe(II)-dependent oxygenases, as well as residues characteristic of the ALKBH subfamily, are conserved between these proteins (Fig. 2).

### Uncoupled Enzymatic Activity of Recombinant ALKBH8 Proteins

**Figure 3:**
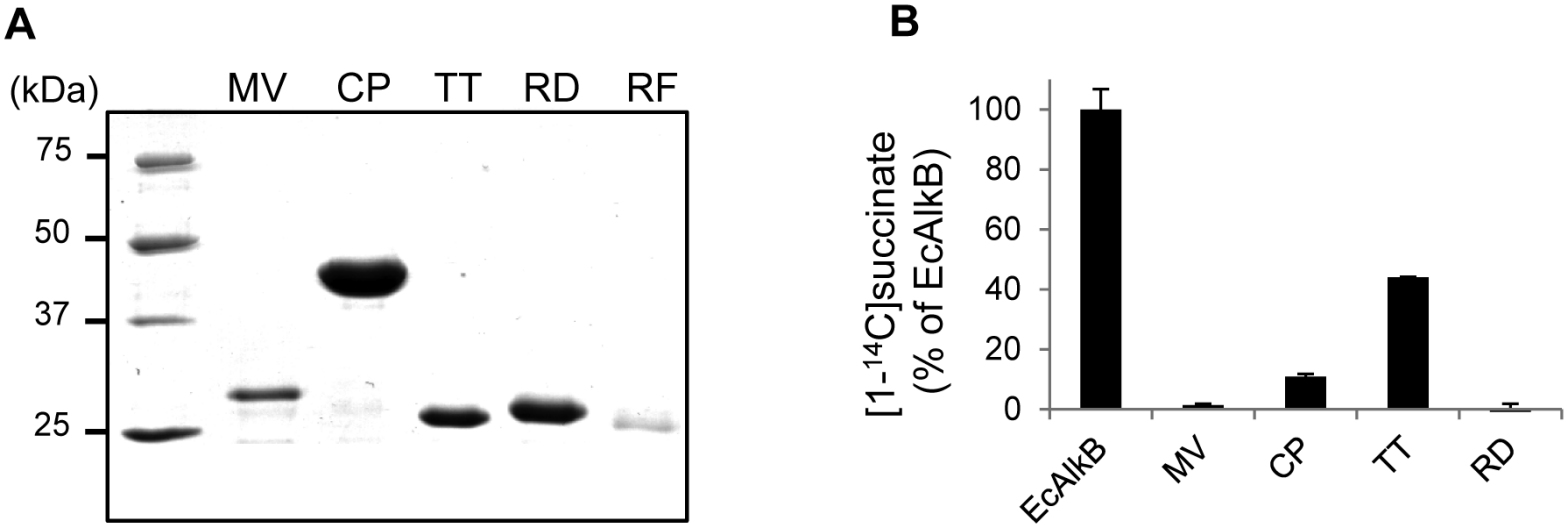
Purification and initial characterization of ALKBH8 proteins. (A) Purification of recombinant His-tagged ALKBH8 proteins used in present study. Proteins were expressed in *E. coli*, purified by metal affinity chromatography and visualized on a Coomassie stained SDS–PAGE gel. (B) Uncoupled activity of recombinant ALKBH8 proteins. [5-^14^C] 2-oxoglutarate was incubated for 30 min at 37°C with 100 pmoles of examined proteins, and remaining [5-^14^C] 2-oxoglutarate was precipitated with 2,4-dinitrophenylhydrazine. The generated [1-^14^C] succinate present in the supernatant was measured by scintillation counting.

Many members of the 2OG/Fe(II)-dependent oxygenase superfamily, including several AlkB proteins, are able to convert 2-oxoglutarate to succinate and carbon dioxide at a low rate in the absence of their true substrate, in the so-called uncoupled reaction. As an initial characterization of the ALKBH8 proteins, we expressed and purified hexahistidine (6xHis) tagged recombinant proteins from *E. coli*, and tested their ability to catalyse the uncoupled reaction. The RF protein, which was expressed and purified at very low yield (Fig. 3A), and the AT protein, which we previously have failed to recover in a soluble form [38], were excluded from this analysis. EcAlkB showed robust uncoupled activity, the TT and CP proteins displayed lower, but significant activity, whereas no activity was detected in the case of the MV and RD proteins (Fig. 3B). These results establish the TT and CP proteins as bona fide members of the 2OG/Fe(II)-dependent oxygenase superfamily, but are inconclusive with respect to the MV and RD proteins, since the extent of uncoupled activity varies between 2OG/Fe(II)-dependent oxygenase superfamily members [48].

### *In vitro* Repair of Site Specific Lesions in DNA by Recombinant ALKBH8 Proteins

**Figure 4:**
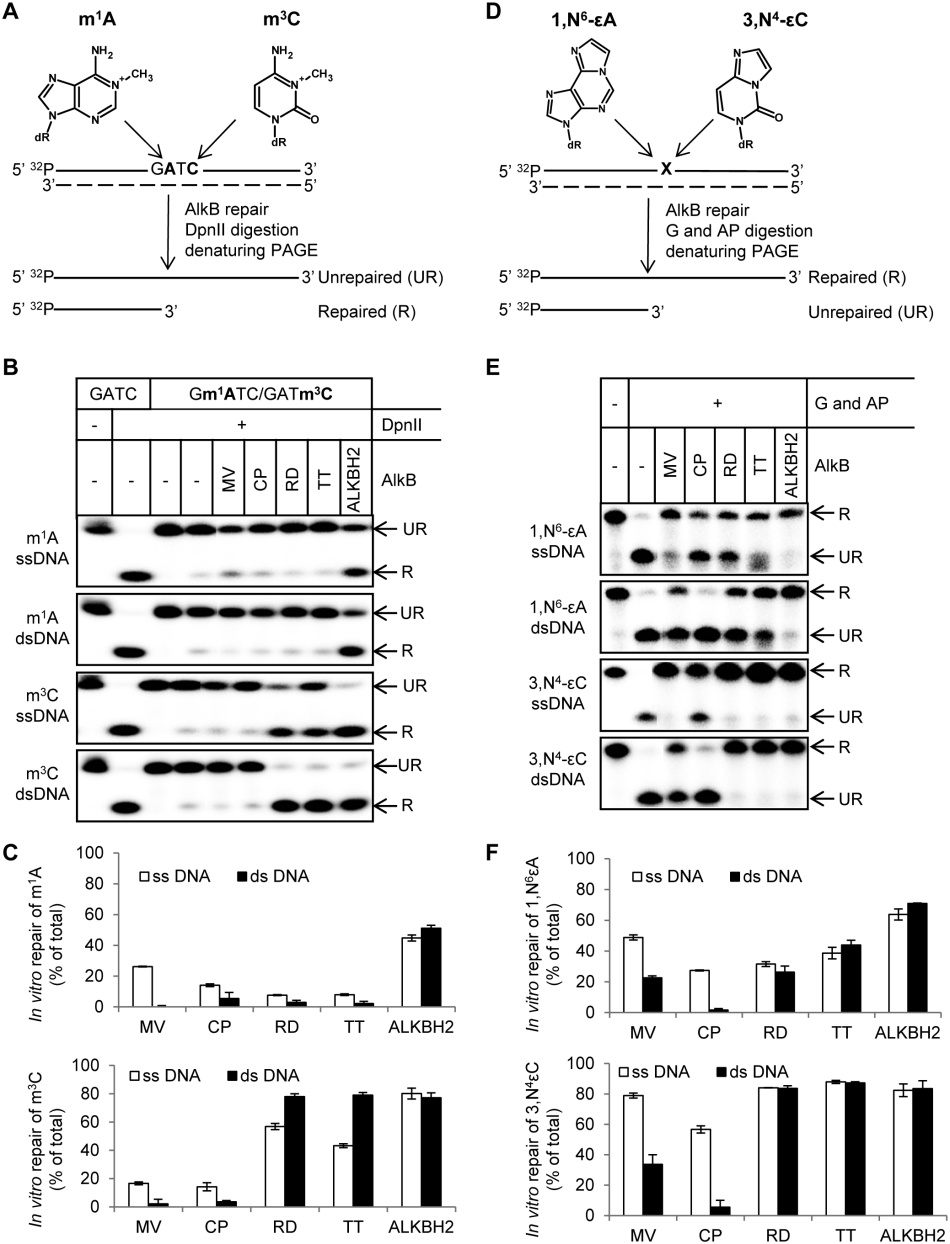
*In vitro* repair activity of ALKBH8 proteins. (A, D) Schematic representation of assay for repair of site specific methyl (A) and etheno lesions (D) in DNA. The dashed line indicates the complementary, lesion-free unlabeled oligonucleotide, which was either present during the repair reaction (dsDNA repair) or added post-repair (ssDNA repair). For repair of methyl lesions, lesion-free oligonucleotide substrates were selectively cleaved by DpnII (A), whereas for etheno adduct repair (D) the lesion-containing base was selectively removed by a glycosylase (G), followed by conversion of the resulting AP site into a single-strand break by an AP endonuclease (AP). (B) Repair activity of purified ALKBH8 proteins on m^1^A and m^3^C in ssDNA and dsDNA. (C) Quantification of results from experiments exemplified in (B). (E) Repair activity of purified ALKBH8 proteins on 1, *N* ^6^-εA and 3, *N* ^4^-εC in ssDNA and dsDNA. The DNA glycosylase ANPG was used on 1, *N* ^6^-εA containing substrates, while Mug was used for substrates with 3, *N* ^4^-εC. (F) Quantification of results from experiments exemplified in (E).

To test the repair activity of the ALKBH8 proteins towards methyl lesions we used oligonucleotides containing a single m^1^A or m^3^C lesion within the recognition sequence (GATC) for the methylation specific restriction enzyme *Dpn*II as substrates [22]. Specifically, these were 25-mer 5′-[^32^P]-end-labeled ssDNA or dsDNA oligomers (the dsDNA substrates contained a lesion-free, unlabeled complementary strand). After incubation with recombinant ALKBH8 protein, the DNA substrates were treated with *Dpn*II to distinguish repaired from unrepaired oligonucleotides, as *Dpn*II cleavage will only occur if the methyl lesion has been removed (Fig. 4A). When ssDNA oligonucleotides were used in the reaction, they were annealed to the complementary (unlabeled) strand after the repair reaction but prior to *Dpn*II digestion, since *Dpn*II will cleave dsDNA but not ssDNA. Human ALKBH2 was included as a positive control for repair of m^1^A and m^3^C. The tested ALKBH8 proteins were unable to repair m^1^A lesions, with the exception of MV AlkB, which showed a very weak repair activity towards m^1^A in ssDNA (Fig. 4B,C). Two of the four tested proteins, RD and TT, exhibited repair activity towards m^3^C in ssDNA and dsDNA (Fig. 4B,C).

Etheno (ε) lesions, such as 1, *N* ^6^-ethenoadenine (1, *N* ^6^-εA), 3, *N* ^4^-ethenocytosine (3, *N* ^4^- εC), *N* ^2^,3-ethenoguanine (*N* ^2^,3-εG), and 1, *N* ^2^-ethenoguanine (1, *N* ^2^-εG) represent exocyclic adducts resulting from the formation of a new imidazole ring on nucleic acid bases, typically induced by lipid peroxidation products or metabolites of vinyl chloride. These highly mutagenic and cytotoxic lesions interfere with normal Watson-Crick base pairing. Although the etheno lesions are repaired mainly through the base excision repair pathway [49], it has been shown that EcAlkB and its human homologues ALKBH2 and ALKBH3 are also able to repair 1, *N* ^6^-εA and 3, *N* ^4^-εC *in vitro* [12], [18], [50]. The simultaneous deletion of ALKBH2, ALKBH3 and the alkyl adenine DNA glycosylase (AAG) in the mouse confers a massively synergistic phenotype after acute inflammation, indicative of overlapping substrate specificities and an *in vivo* role for repairing etheno adducts [51]. It has also been shown that some bacterial AlkB proteins efficiently repair etheno adducts, while having low or no activity on methylated bases [38]. To test the ability of the ALKBH8 proteins to repair etheno adducts *in vitro*, they were incubated with 5′-[^32^P]-end-labeled oligonucleotides containing 1, *N* ^6^-εA or 3, *N* ^4^-εC. To assess whether repair has occurred, the oligonucleotides were treated with a DNA glycosylase which will only cleave the substrate if the lesion is intact, *i.e.* a conceptually opposite approach to that of using *Dpn*II, and one which does not require a particular sequence at the lesion site (Fig. 4D). For cleavage of 1, *N* ^6^-εA containing substrates, the DNA glycosylase ANPG (also known as AAG or MPG) was used, while Mug was used for substrates with 3, *N* ^4^-εC. After treatment with DNA glycosylase, strand-breaks were introduced at resulting abasic sites through cleavage with the human AP endonuclease 1 (Fig. 4D). When single-stranded oligonucleotides were used in the AlkB reaction, they were annealed to their complementary strand prior to DNA glycosylase treatment. Again, human ALKBH2 was included as a positive control. The four recombinant ALKBH8 proteins (MV, CP, RD, TT) showed strong repair activity towards 3, *N* ^4^-εC and somewhat weaker activity towards 1, *N* ^6^-εA *in vitro* (Fig. 4E,F). The MV protein was more active on ssDNA compared to dsDNA, whereas the CP protein repaired 3, *N* ^4^-εC and 1, *N* ^6^-εA only in ssDNA (Fig. 4E,F). In summary, the tested ALKBH8 proteins displayed *in vitro* repair activity on DNA, and they are generally more active on etheno adducts than on methyl lesions, similarly to the *R. etli* ALKBH8 protein investigated previously [38].

### ALKBH8-mediated Repair of Chemically Induced Methyl and Etheno Lesions *in vivo*

**Figure 5:**
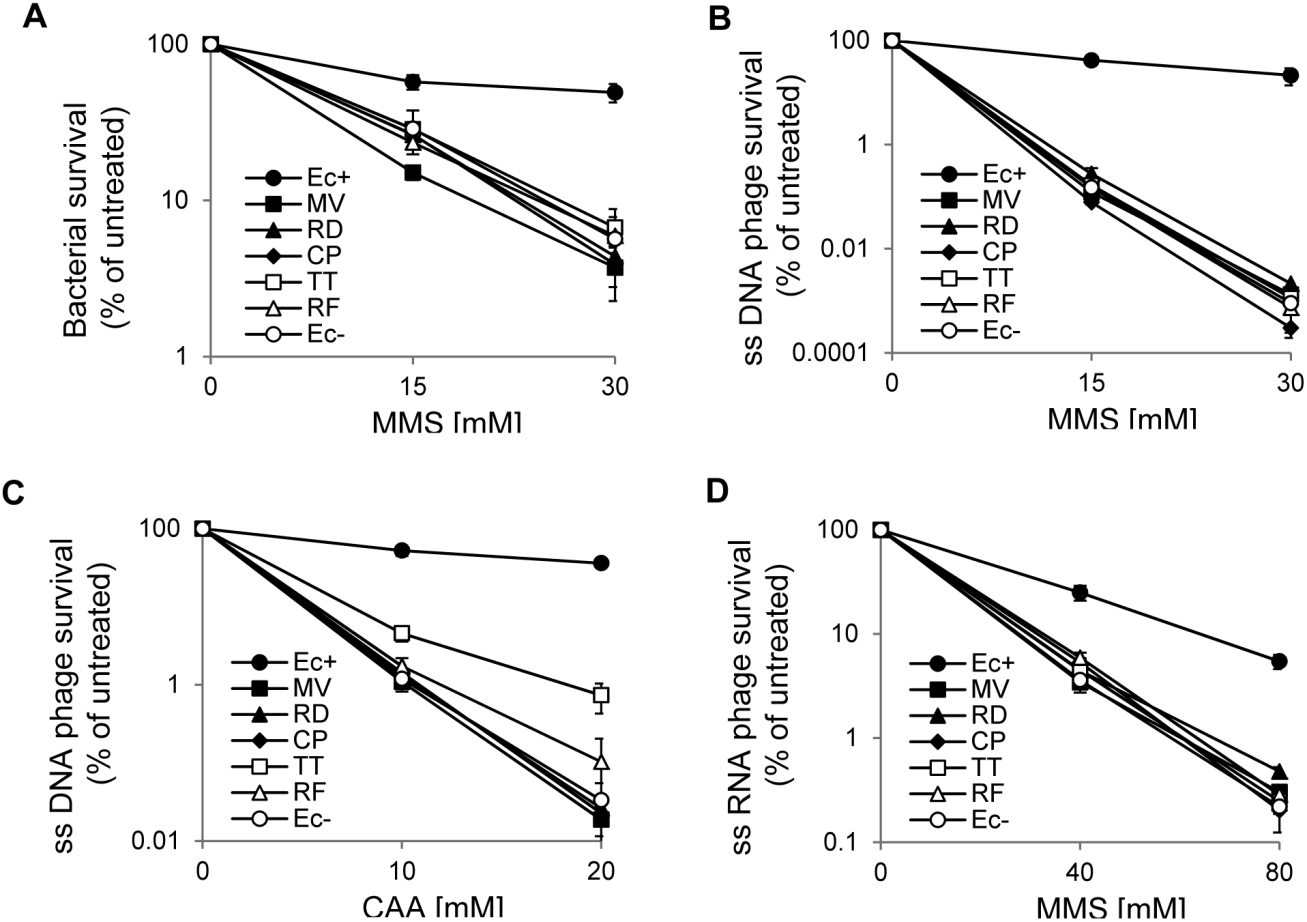
ALKBH8 mediated repair of MMS- or CAA induced lesions in *E. coli.* AlkB-deficient (*alkB*) *E. coli* carrying either an empty expression plasmid (Ec–), or corresponding plasmids for expression of the indicated ALKBH8 proteins or EcAlkB (Ec+) were used in all experiments. (A) MMS-sensitivity of bacteria. (B) survival of MMS-treated ssDNA phage M13. (C) survival of CAA-treated ssDNA phage M13. (D) survival of MMS-treated ssRNA phage MS2.

*E. coli alkB* mutants are sensitive to the methylating agent methyl methanesulfonate (MMS), due to their inability to repair replication blocking lesions, such as m^1^A and m^3^C. Such lesions are introduced at a particularly high frequency in ssDNA, relative to dsDNA. Consequently, when infected by MMS-treated ssDNA bacteriophage, *alkB* mutants show a dramatically reduced ability to generate progeny phage, as they are unable to reactivate the damaged phage DNA through removal of deleterious methyl lesions [52]. To examine if the ALKBH8 proteins were able to complement the MMS-sensitive phenotype, they were expressed in AlkB-deficient bacteria, which were subsequently exposed to MMS and their survival assessed. While the expression of EcAlkB complemented the MMS-sensitive phenotype of the mutant bacteria, none of the ALKBH8 proteins had this effect (Fig. 5A). Similarly, only EcAlkB was able to increase the reactivation of the MMS-treated ssDNA phage M13 (Fig. 5B).

To test the ability of the ALKBH8 proteins to repair etheno adducts, they were expressed in *alkB E. coli*, and their ability to reactivate chloroacetaldehyde (CAA)-treated ssDNA phage M13 was measured. CAA causes the formation of exocyclic DNA adducts with the following relative efficiencies: 1, *N* ^6^-εA>3, *N* ^4^-εC>*N* ^2^,3-εG>1, *N* ^2^-εG [53], [54]. The majority of the ALKBH8 proteins were unable to reactivate CAA-treated ssDNA phage; only the TT (*T. thermophila*) and RF (*Rickettsia felis*) proteins caused a modest increase in progeny phage formation, but the effect was substantially lower than that observed for EcAlkB (Fig. 5C).

Certain AlkB proteins are able to repair methyl lesions in RNA *in vitro*, and can reactivate MMS treated RNA phage when expressed in AlkB-deficient *E. coli* [9], [11], [37]. To test the activity of the ALKBH8 proteins towards RNA, *alkB E. coli* expressing these proteins were infected with MMS-treated RNA phage MS2. While overexpression of the EcAlkB protein substantially increased the survival of MMS-treated phage MS2, this was not the case for any of the ALKBH8 proteins (Fig. 5D), indicating that they are not RNA repair proteins.

These *in vivo* complementation experiments indicate, similarly to the *in vitro* repair assays, that ALKBH8 proteins prefer etheno adducts over methyl lesions, but they also indicate that these enzymes do not efficiently repair canonical EcAlkB substrates.

### MMS/CAA Sensitivity and tRNA^Gly(UCC)^ Modification Status of ALKBH8-deficient *Agrobacterium tumefaciens*

In our previous study, the ALKBH8 protein from the bacterium *R. etli* showed robust repair activity on etheno adducts [38]. On the other hand, the bacterial ALKBH8 proteins display a high degree of sequence similarity to human ALKBH8 not only in the core oxygenase domain, but also in the so-called nucleotide recognition lid region (Fig. 2), pointing towards a role in tRNA modification. Human and plant ALKBH8 are both involved in wobble uridine modification of tRNA^Gly(UCC)^, and the sequence of this tRNA is rather well conserved from humans to bacteria, especially in the anticodon loop, which is identical. To address the potential role of bacterial ALKBH8 proteins in tRNA modification, we decided to investigate the wobble uridine modification status of tRNA^Gly(UCC)^ in ALKBH8-deficient versus wild-type bacteria. For this purpose, we selected *Agrobacterium tumefaciens,* which can be subjected to gene knock-out by the so-called TargeTron technology [44], [45].

**Figure 6:**
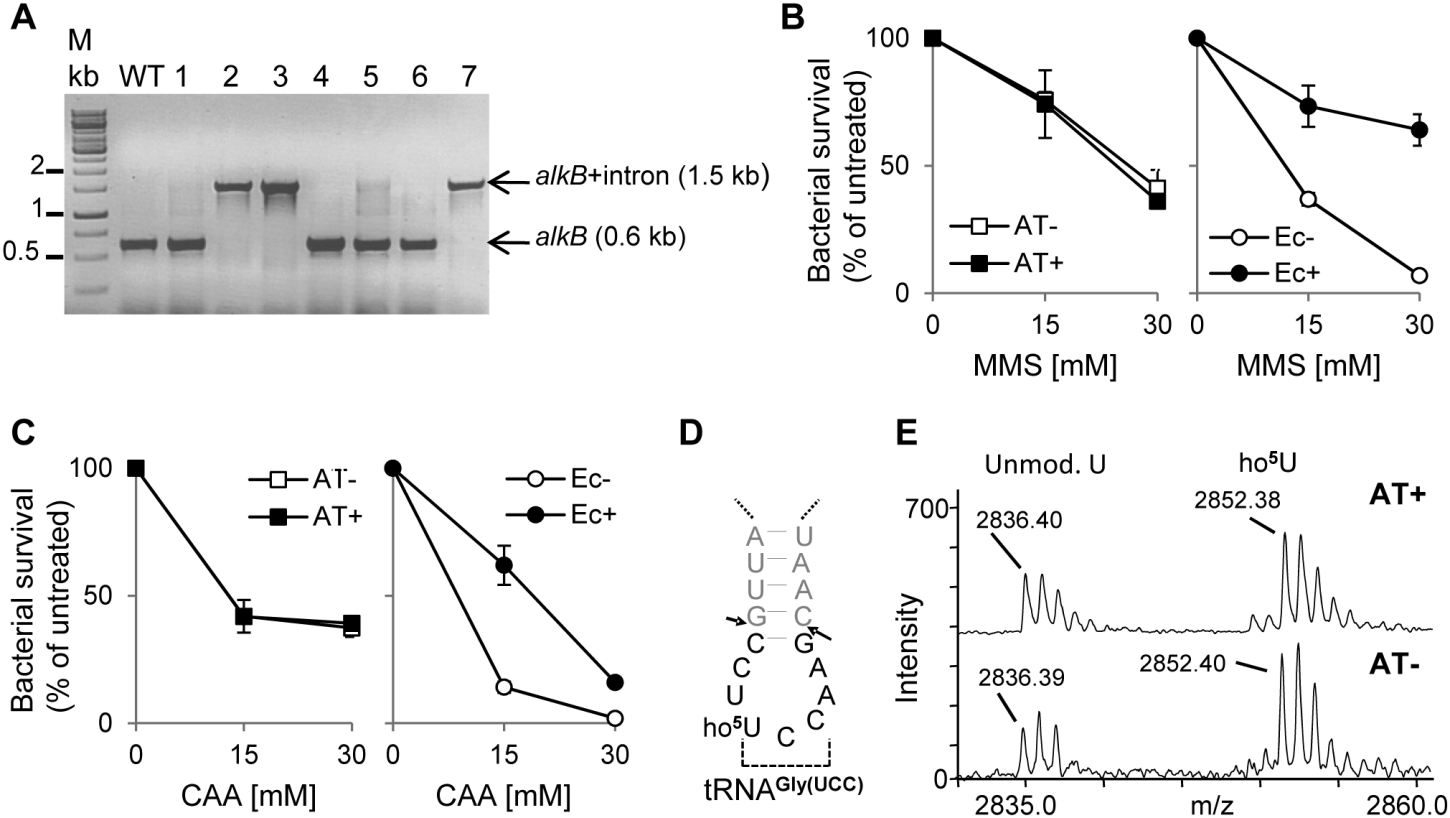
Generation and characterization of AT-deficient *Agrobacterium tumefaciens*. (A) Inactivation of the *A. tumefaciens alkB* (*AT*) gene by site specific intron insertion. After intron induction, bacteria were plated and resulting colonies were subject to colony PCR using *alkB* (*AT*) specific primers. The lower arrow indicates the 0.6 kb fragment resulting from the non-disrupted gene (colonies 1, 4, 5, and 6), while the upper arrow indicates 1.5 kb fragment generated from the *alkB* gene disrupted by intron integration (colonies 2, 3, and 7). (B) MMS sensitivity of AT-deficient (AT–) versus AT-proficient (AT+) *A. tumefacies.* Bacteria were incubated in the presence of the indicated concentrations of MMS, then plated on agar plates, and survival scored by colony counting. *E. coli* served as control. (C), CAA sensitivity of AT-deficient (AT–) versus AT-proficient (AT+) *A. tumefacies.* Same as (B), but CAA was used instead of MMS. (D) Anticodon stem-loop of tRNA^Gly(UCC)^ from *A. tumefaciens*. Black print indicates the anticodon-containing fragment generated by cleavage with RNase T1 (at arrows). (E) Wobble uridine modification status of tRNA^Gly(UCC)^ from wild-type and *alkB* (*AT*) mutant *A. tumefaciens*. MALDI-TOF MS spectra of the anticodon-containing RNase T1 fragment illustrated in (D) are shown, and measured masses indicated. Calculated masses for the unmodified and ho^5^U modified versions of the fragment (CCUUCCAAG) are 2836.37 and 2852.37, respectively (the masses refer to fragments with 2′–3′ cyclic phosphate termini, which represent the major digestion products).

When using the TargeTron technology, the gene of interest can be disrupted by site specific insertion of a redesigned Group II intron [44], [45]. We found the ALKBH8-encoding gene to be efficiently targeted; 3 out of 7 clones tested by colony-PCR carried the inserted intron (Fig. 6A). As the majority of bacterial AlkB proteins appear to be DNA repair enzymes, it was first investigated if disruption of the *A. tumefaciens alkB* gene caused sensitivity towards the genotoxic agents MMS and CAA. AlkB-deficient and wild-type *A. tumefaciens* bacteria were similarly sensitive as to MMS and CAA treatments, whereas AlkB-deficient *E. coli,* as expected and previously reported, were more sensitive to treatment with these DNA damaging agents than bacteria expressing EcAlkB (Fig. 6B,C). These results showed that the AT protein does not protect *A. tumefaciens* against the tested DNA damaging agents, suggesting that the AT protein does not play an important role in repair of methyl and etheno lesions.

To assess the wobble uridine modification status, MALDI-TOF mass spectrometry analysis was performed on RNase T1 digested tRNA^Gly(UCC)^ isolated from *A. tumefaciens*. The data indicated that *A. tumefaciens* tRNA^Gly(UCC)^ contains 5-hydroxyuridine (ho^5^U) at the wobble position, as an RNase T1 fragment containing the anticodon displayed a mass increase of 16 Da relative to the unmodified sequence (Fig. 6D,E), a result compatible with the action of a hydroxylase such as ALKBH8. However, this uridine modification was also present in the ALKBH8-deficient bacteria, showing that ALKBH8 is not involved in wobble uridine modification of tRNA^Gly(UCC)^, and suggesting that this may also be the case for other bacterial ALKBH8 proteins.

### ALKBH8-mediated Hydroxylation of mcm^5^U in tRNA

**Figure 7:**
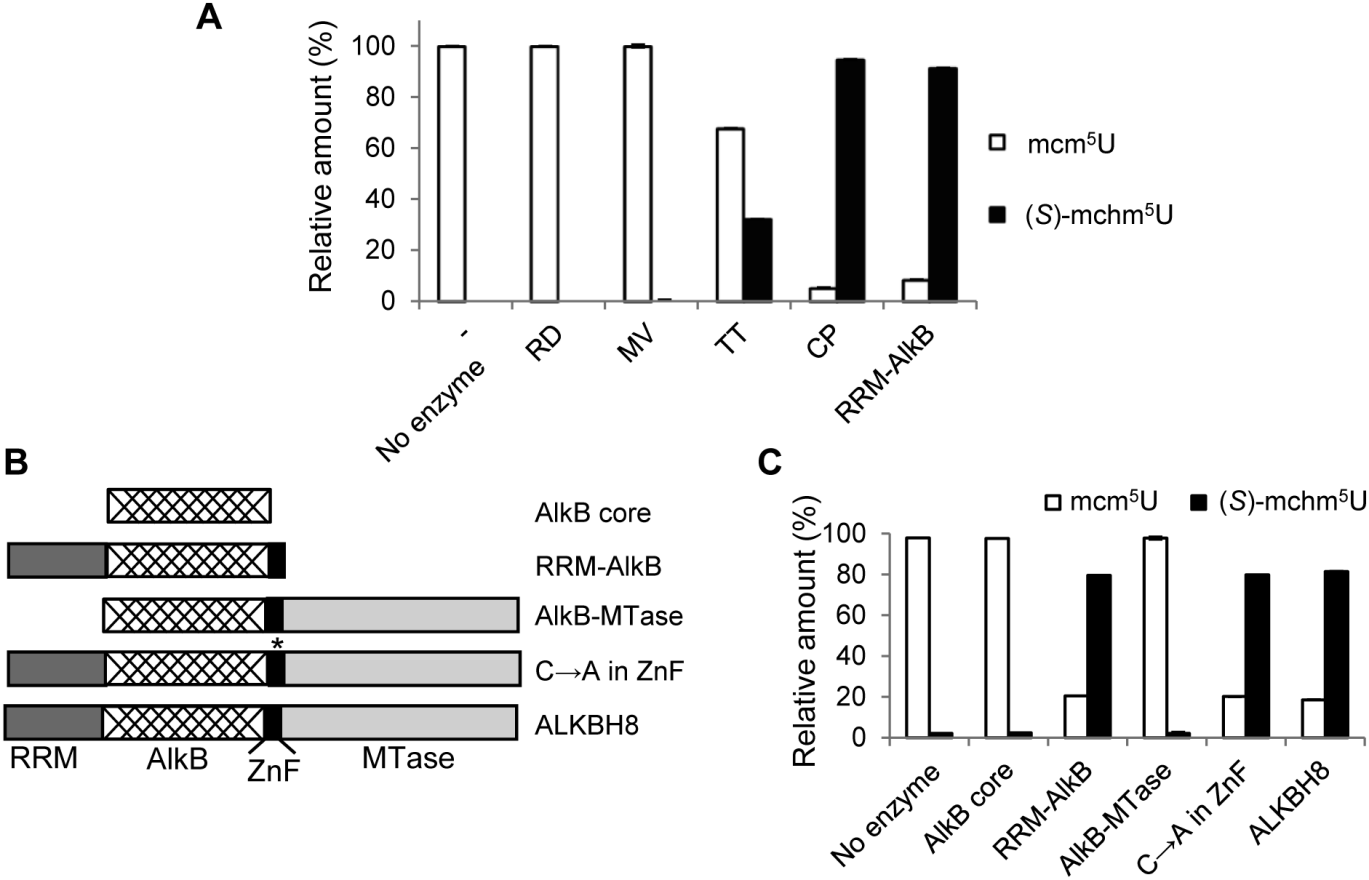
*In vitro* analysis of tRNA modifying activity of ALKBH8 proteins. (A) tRNA modifying activity of ALKBH8 proteins from various organisms. The indicated proteins were incubated with *S. cerevisiae* tRNA, and the ability of enzymes to catalyze the conversion of mcm^5^U to (*S*)-mchm^5^U was investigated by LC-MS/MS analysis of tRNA nucleosides. The RRM-AlkB part of human ALKBH8 was used as positive control. (B, C) Analysis of the tRNA modifying activity of deletion and point mutants of human ALKBH8. (B) Overview of tested proteins. “C→A in ZnF” refers to a mutant where the three conserved Cys residues (Cys341, Cys 343 and Cys 349) of the ZnF moiety have been replaced by alanine. (C) The indicated proteins were incubated with mcm^5^U containing tRNA from the so-called KI(MT^+^) mouse, *i.e.* a gene-targeted mouse expressing the ALKBH8 MTase, but not the oxygenase (AlkB) activity. Enzymatic conversion of mcm^5^U to (*S*)-mchm^5^U was investigated by LC-MS/MS analysis of tRNA nucleosides.

Mammalian and plant ALKBH8 specifically hydroxylate mcm^5^U into (*S*)-mchm^5^U at the wobble position of the anticodon in tRNA^Gly(UCC)^ (Fig. 1C) [33], [34]. As *S. cerevisiae* lacks an ALKBH8 orthologue, yeast tRNA^Gly(UCC)^ contains wobble mcm^5^U, and total yeast tRNA is thus a suitable substrate for testing the potential mcm^5^U hydroxylating ability of ALKBH8 proteins [34]. Yeast tRNA was incubated with various ALKBH8 enzymes in the presence of appropriate cofactors, and then enzymatically digested to nucleosides, which were analyzed by LC–MS/MS. The RRM/AlkB/ZnF portion of human ALKBH8 (RRM-AlkB; aa 1–354) was included as a positive control. Only for the two eukaryotic, protozoan ALKBH8 proteins, CP and TT, was conversion of mcm^5^U to (*S*)-mchm^5^U observed (Fig. 7A). These results suggest that the ALKBH8 proteins from the protozoa *C. parvum* and *T. thermophila* are involved in biosynthesis of wobble (*S*)-mchm^5^U.

### Enzymatic Activity of Deletion Mutants of Human ALKBH8

The CP protein was the most active out of the two protozoan ALKBH8 proteins that showed tRNA modification activity, and also contained all three domains found in plant ALKBH8, *i.e.* RRM/AlkB/ZnF. The less active TT protein, in contrast, only consisted of an AlkB moiety, suggesting that the RRM and ZnF domains may contribute positively to ALKBH8 activity. To investigate this, we tested the tRNA modifying activity of various mutants of human ALKBH8, containing deletions or point mutations in these domains, depicted in Fig. 7B. As substrate in these assays, we used tRNA from a gene-targeted mouse (denoted KI(MT^+^)) expressing the MTase activity, but not the oxygenase activity of ALKBH8, thereby showing an accumulation of mcm^5^U [34]. We found that changing three of the conserved cysteine residues to alanine in the ZnF domain did not affect ALKBH8 activity, indicating that this structure is not crucial for ALKBH8 activity (Fig. 7C). In contrast, the two mutants lacking the RRM domain (“AlkB core” and “AlkB-MTase”) were devoid of enzymatic activity, indicating the importance of this domain.

## Discussion

While ALKBH8 proteins from mammals and plants have been established as tRNA modification enzymes, ALKBH8 from the bacterium *R. etli* was shown to possess repair activity towards etheno adducts in DNA [33], [34], [38], [39]. Based on this apparent duality of the ALKBH8 proteins, we have here investigated such proteins from a wide range of species, both with respect to DNA/RNA repair and tRNA modification activities, and the results are summarized in Table 1. The major findings in our study were that DNA repair activity actually could be detected *in vitro* for all tested proteins, and that two ALKBH8 proteins, originating from protozoa, also displayed tRNA modification activity similar to that of ALKBH8 from multicellular eukaryotes.

**Table 1:** Summary of experiments.

Protein	*In vivo* experiments		*In vitro* experiments								
	ssDNA(MMS)	ssDNA(CAA)	ssDNA	dsDNA	ssDNA	dsDNA	ssDNA	dsDNA	ssDNA	dsDNA	tRNA
	ssRNA(MMS)		m^1^A	m^1^A	m^3^C	m^3^C	εA	εA	εC	εC	mcm^5^U
	Survival(MMS)										
RF	−	+	*	*	*	*	*	*	*	*	*
RD	−	−	−	−	++	++	+	+	++	++	−
TT	−	+	−	−	+	++	+	+	++	++	+
MV	−	−	+	−	−	−	++	+	++	+	−
CP	−	−	−	−	−	−	+	−	+	−	++

++, activity comparable to positive control (PC); +, substabtially lower activity than PC (>30% reduction); −, no detectable activity;

*, unable to produce recombinant protein.

Many enzymes display promiscuous activities *in vitro*, *i.e.* activities that are different from the one for which the enzyme evolved, and that have no physiological role [55]. It is believed that such promiscuity may play an important part in the evolution of novel enzyme functions through gene duplication and mutation. Gene duplication allows for the retention of the original activity by one gene copy, whereas the other copy can be subjected to optimization of the promiscuous activity through amino acid substitution, until the promiscuous activity actually becomes a physiologically beneficial and selectable trait. Bacteria typically have 1–2 AlkB proteins, and the vast majority (>90%) of these appear to be DNA repair enzymes, whereas multicellular eukaryotes typically have several ALKBH proteins, most of which have other roles, *e.g.* in RNA modification. Thus, it is rather likely that the ALKBH family of enzymes in eukaryotes has evolved from an ancestral DNA repair enzyme, and it is not very surprising that some DNA repair activity can be detected *in vitro* for ALKBH proteins involved in other processes. AlkB proteins that function in DNA repair, such as the founding member *E. coli* AlkB, are in themselves rather promiscuous, as they can repair a wide range of DNA adducts (methyl, etheno, ethano, etc) on several different nucleobases independent of sequence context. In contrast, the tRNA modification activity of ALKBH8 is much more specific, as it appears to exclusively occur on a single mcm^5^U-containing tRNA species, tRNA^Gly(UCC)^. We therefore believe that the tRNA modification activity observed with the protozoan CP and TT proteins *in vitro* reflect their true, physiologically relevant function, whereas the detected repair activity represents a “ghost” of an evolutionary precursor, now manifested as a promiscuous activity. Indeed, a similar promiscuity has previously been observed for the mammalian ALKBH1 proteins, which show activity on methyl lesions in ssDNA and ssRNA as well as on histone proteins [26], [56].

Two of the ALKBH8 proteins studied here, TT and CP, both of which originate from protozoa, catalyzed the hydroxylation of mcm^5^U into (*S*)-mchm^5^U in tRNA. The observed activity of the CP protein is not very surprising, as this protein has a RRM/AlkB/ZnF architecture also found in the tRNA-modifying ALKBH8s from mammals and plants. The importance of the RRM and ZnF domains in human ALKBH8 was recently demonstrated; the RRM domain provides affinity towards RNA (whereas the AlkB domain does not contribute to RNA binding), and the ZnF moiety increases the overall stability of the protein [57]. Accordingly, we observed that deletion of the RRM moiety abolished the activity of human ALKBH8, whereas mutation of the conserved Cys residues of the ZnF moiety had no effect. Therefore, the observed activity of the TT protein was somewhat unexpected, as this protein solely consists of an AlkB domain. However, this result gives important clues regarding the evolution of the tRNA modifying ALKBH8 function: point mutations in an ancestral repair protein may have yielded a beneficial, but suboptimal tRNA modifying activity, followed by the acquisition of RRM and ZnF domains, giving improved substrate affinity and enzyme stability, respectively. Finally, the fusion between the RRM/AlkB/ZnF moiety and a Trm9-like methyltransferase in animals has likely further improved the efficacy of the modification system by providing a direct channeling of mcm^5^U-modified tRNA^Gly(UCC)^ from the ALKBH8 methyltransferase to the hydroxylase.

We have here generated an *A. tumefaciens* mutant with an inactivated ALKBH8 gene and found the modification status of tRNA^Gly(UCC)^ to be unaltered relative to the wild-type bacterium. This clearly suggests that bacterial ALKBH8s, unlike their eukaryotic counterparts, do not target the wobble uridine of tRNA^Gly(UCC)^. However, the close sequence similarity between bacterial and eukaryotic ALKBH8s in the so-called nucleotide lid region (NRL), which is responsible for interaction with the nucleic acid substrate, may still suggest that bacterial ALKBH8s are RNA modification enzymes. Also, our demonstration of tRNA modification activity for the TT protein, which, like the bacterial ALKBH8s, lacks the RRM- and ZnF-domains, supports this notion. We have previously studied the ALKBH8 protein from the bacterium *R. etli,* and found it to be as efficient as *E. coli* AlkB in reactivating CAA-treated (etheno adduct-containing) ssDNA phage [38]. In contrast, the two bacterial ALKBH8 proteins studied here, RD and RF, showed substantially lower (RF) or negligible (RD) repair activity, and also considerably lower than for the protozoan TT protein, which also displayed tRNA modifying activity. Moreover, it should be noted that the *R. etli* ALKBH8 protein, when compared with the RD and RF proteins, is less similar to eukaryotic ALKBH8s, and lacks several of the conserved residues shared between other ALKBH8s (Fig. 2). Based on the above, we favor the notion that the “canonical” bacterial ALKBH8s such as RF and RD are RNA modification enzymes targeting a (yet unidentified) substrate resembling the mcm^5^U moiety recognized by the eukaryotic ALKBHs.

The present work provides important insights on the ALKBH8 proteins, but many key questions remain unanswered. It will be of great interest to reveal the physiologically relevant substrate(s) of the bacterial ALKBH8 proteins. Here, the *A. tumefaciens* mutant described in the present work may represent a useful tool. Conceivably, a systematic, global analysis of the RNA modification pattern in the mutant versus wild-type bacteria may uncover this substrate. Furthermore, we have here shown that the ALKBH8 protein (TT) from *Tetrahymena thermophilus*, unlike its mammalian counterpart, is able to catalyse mcm^5^U hydroxylation even in the absence of an RRM domain. This indicates that its AlkB domain (catalytic moiety) has an intrinsic affinity for the tRNA substrate, suggesting that this protein may be particularly well suited for structural studies aimed at solving the structure of an enzyme/substrate complex, thereby yielding insights on the detailed ALKBH8 mechanism.

## Supporting Information

Table S1 **Oligonucleotides used in the present study.**
(PDF)
