# Supplementary material for: Protozoan ALKBH8 Oxygenases Display both DNA Repair and tRNA Modification Activities
Source: PLoS One. 2014 Jun 10;9(6):e98729. doi: 10.1371/journal.pone.0098729 (PMC4051686; doi:10.1371/journal.pone.0098729)
Supplement: Table S1 — Oligonucleotides used in the present study. (PDF) [file pone.0098729.s001.pdf]

Zdżalik et al. **Table S1. Oligonucleotides used in the present study.**

Purpose	Name	Sequence (5'–3')	Additional information
Cloning of the coding sequences of AlkB from *Acanthamoeba polyphaga mimivirus* into pET-28a(+) and pJB658 plasmids	MV-F	GGCAGACATATGATGTCTAAAAAGTTCA AAATG	Restriction sites are underlined.
	MV-R	CGAGGTGGATCCTTAATTAGGAGATCTT TTTG	
Cloning of the coding sequence of AlkB from *Roseobacter denitrificans OCh 114* into pET-28a(+) and pJB658 plasmids	RD-F	TTCTGGCATATGATGCGGTCAGCCCACC TCTC	
	RD-R	AGGCAAGGATCCTTAAAACTTCATCGTC CGAA	
Cloning of the coding sequence of AlkB from *Rickettsia felis URRWXCal2* into pET-28a(+) and pJB658 plasmids	RF-F	CCTATCGCTAGCATGAGTCAACTTAGTC TTTT	
	RF-R	GGCACGGATCCTTACAAAATCGCTTTTC TAA	
Cloning of the coding sequence of AlkB from *Cryptosporidium parvum Iowa II* into pET-28a(+) and pJB658 plasmids	CP-F	ACTACTGGCATATGACAGAAGAGATCAT TTACAGC	
	CP-R	ATGGATCCTTATATTCTTTCAGGCAAAA TTCTAATAG	
Cloning of the coding sequence of AlkB from *Tetrahymena thermophila* into pET-28a(+) and pJB658 plasmids	TT-1F	GGTCTACATATGGATGACCAAAAACAAA AACTTTTTG	
	TT-1R	ATGGATCCTCAAGCATCAACTAAAGCTT TTCTG	
	TT-2F	CACAGACATTCGATCAGGTACAGGGTTT GAG	Oligos to mutate all glutamine coding TAG codons to CAG and the glutamine coding TAA codons to CAA.
	TT-2R	GTACCTGATCGAATGTCTGTGCTGAATC	
	TT-3F	GGAGAACTCAACATTATGG	
	TT-3R	CCATAATGTTGAGTTCTCC	
	TT-4F	AAAGTTATGCCAGATCAAATGATTATTA ACGAGTATTTACCAGGTCAGGGTATC	
	TT-4R	CATTTGATCTGGCATAACTTTTATAACT TTATCATCTATCAGTCTTTGGCAGAAAT TG	
	TT-5F	GAAAGACTATTCAGAGGAGTAC	
	TT-5R	GTACTCCTCTGAATAGTCTTTC	
Construction of *Agrobacterium tumefaciens* C58 *alkB* null mutant with TargeTron Gene Knockout System (Sigma Aldrich).	EBS	CGAAATTAGAAACTTGCGTTCAGTAAAC	Universal Primer from the TargeTron Gene Knockout System
	IBS	AAAAAAGCTTATAATTATCCTTAATCGT CTCGATAGTGCGCCCAGATAGGGTG	Specific primers designed by The TargeTron algorithm available on the producer's website: http://www.sigma-genosys.com/targetron
	EBS1d	CAGATTGTACAAATGTGGTGATAACAGA TAAGTCTCGATAAGTAACTTACCTTTCT TTGT	
	EBS2	TGAACGCAAGTTTCTAATTTCGATTACG ATTCGATAGAGGAAAGTGTCT	
	AlkBAT-F	ACTACTGGCATATGGATTGCGTCGCTAA TCCAACTCTTC	Primers specific for *Agrobacterium tumefaciens* C58 *alkB* gene used to screen for knockouts in the colony PCR
	AlkBAT-R	ATGTAGACGGATCCTCAGGGCCGTTTCA AACCCAGG	
m^1A repair *in vitro*	m^1A	ATTCTCGTTAGGm^1ATCGCGTCAAGCC	Restriction sites recognized by *Dpn*II is underlined
m^3C repair *in vitro*	m^3C	ATTCTCGTTAGGATm^3CGCGTCAAGCC	
1,N^6-εA repair *in vitro*	1,N^6-εA	GCTACCTACCTAGCGACCTεACGACTGT CCCACTGCTCGAA	
3,N^4-εC repair *in vitro*	3,N^4-εC	ATTCTCGTTAGGATεCGCGTCAAGCC	
$tRNA^{Gly(UCC)}$ isolation from *A. tumefaciens*		AGCGGGTAGCGGGAATCGAACCCGCGTT AT	3′-biotinylated
Removal of RRM domain from ALKBH8	446	ACTACTGGCATATGGGGAGGCCTCAAGC CTTACCACC	Restriction sites are underlined.
	53	ATGTAGACGTCGACTCAGGCCTTTTGAA GAATCACACACC	
Removal of RRM domain from RRM-AlkB	446	ACTACTGGCATATGGGGAGGCCTCAAGC CTTACCACC	Restriction sites are underlined.
	78	ATGTAGACGTCGACTCACCTCACTTTCC TAAATGTAAATGATGT	
Substitutions Cys341Ala, Cys343Ala, Cys349Ala in ALKBH8	446	ACTACTGGCATATGGACAGCAACCATCA AAGTAATTAC	Restriction sites are underlined.
	358	GACCAACGGGTAGCTAGCGTTAGCAGGT GTTTGCC	
	359	TAGCTACCCGTTGGTCGCTGATAGCCAG AGG	
	53	ATGTAGACGTCGACTCAGGCCTTTTGAA GAATCACACACC	
